# Dense 3D Point Cloud Environmental Mapping Using Millimeter-Wave Radar

**DOI:** 10.3390/s24206569

**Published:** 2024-10-12

**Authors:** Zhiyuan Zeng, Jie Wen, Jianan Luo, Gege Ding, Xiongfei Geng

**Affiliations:** 1 China Waterborne Transport Research Institute, Beijing 100088, China; zengzhiyuan@wti.ac.cn (Z.Z.);; 2 School of Electronic Information, Wuhan University, Wuhan 430072, China

**Keywords:** millimeter-wave radar, radar point cloud processing, radar mapping, convolutional neural network

## Abstract

To address the challenges of sparse point clouds in current MIMO millimeter-wave radar environmental mapping, this paper proposes a dense 3D millimeter-wave radar point cloud environmental mapping algorithm. In the preprocessing phase, a radar SLAM-based approach is introduced to construct local submaps, which replaces the direct use of radar point cloud frames. This not only reduces data dimensionality but also enables the proposed method to handle scenarios involving vehicle motion with varying speeds. Building on this, a 3D-RadarHR cross-modal learning network is proposed, which uses LiDAR as the target output to train the radar submaps, thereby generating a dense millimeter-wave radar point cloud map. Experimental results across multiple scenarios, including outdoor environments and underground tunnels, demonstrate that the proposed method can increase the point cloud density of millimeter-wave radar environmental maps by over 50 times, with a point cloud accuracy better than 0.1 m. Compared to existing algorithms, the proposed method achieves superior environmental map reconstruction performance while maintaining a real-time processing rate of 15 Hz.

## 1. Introduction

In the context of the rapid development of application fields such as autonomous driving [1] and virtual or augmented reality [2], the requirements for environmental perception by robots are increasingly demanding in terms of accuracy, resolution, dimensionality, and scale. In recent years, high-resolution multi-dimensional environmental reconstruction technology oriented toward scenarios has become a hot topic of research. The construction of environmental maps for large scenes can generally be based on SLAM technology, which combines the sensor data and motion model of the robot to estimate the robot’s pose and the structure of the environment in real time and continuously optimizes the map construction and self-localization results.

Existing robotic systems are typically equipped with a variety of sensors to acquire multi-dimensional information about the surrounding environment. The primary sensors include a camera, LiDAR, and millimeter-wave radar. The camera, with its rich color and texture perception capabilities, possesses the ability to construct detailed environmental maps, and its low cost has led to widespread application. However, a camera is susceptible to the influence of external lighting, weather, and other factors. LiDAR offers high resolution in terms of distance and angle, providing dense and accurate three-dimensional point cloud data, and is not limited by lighting conditions, making its environmental mapping capabilities relatively stable. Nevertheless, LiDAR cannot penetrate environments with smoke, dust, or fog, and its environmental mapping capabilities may be limited when dealing with surfaces of low reflectivity or transparent objects. Millimeter-wave radar provides a longer detection range and excellent penetration capabilities, adapts to various weather conditions, and features velocity measurement, which has garnered widespread attention for environmental mapping based on millimeter-wave radar in recent years [3].

According to the different working modes of millimeter-wave radar systems used for environmental reconstruction, millimeter-wave radar can mainly be divided into the Transmit Beamforming Mode and Received Beamforming Mode (also known as Multiple-Input Multiple-Output (MIMO) Mode). The working principle of the Transmit Beamforming Mode is similar to that of LiDAR, which synthesizes narrow beams by controlling the phase of multiple transmitting antennas and scans the spatial azimuth through either phase or mechanical scanning to obtain high-resolution radar images [4]. In [5], the results of environmental map construction using radar SLAM in indoor and outdoor environments using the Transmit Beamforming Mode are reported. In [6], the robustness of environmental map construction using Transmit Beamforming Mode radar was validated in extreme weather conditions, such as fog and snow. However, Transmit Beamforming Mode radar generally achieves omnidirectional scanning through mechanical rotation, resulting in lower imaging frame rates. Additionally, rapid movement of the radar platform can distort the imaging results, and high costs further limit its practical applications. Received Beamforming Mode radar typically employs orthogonal transmission with multiple transmitting antennas and simultaneous reception using multiple receiving antennas, achieving fast imaging through algorithms like digital beamforming. It offers higher imaging frame rates and lower costs, making it the mainstream radar system for vehicular applications. There are various methods for implementing SLAM or environmental map construction with MIMO radar. In [7], a method for environmental map construction based on Doppler information is proposed, while in [8], a method based on inter-frame matching is introduced. However, current environmental map construction using MIMO millimeter-wave radar faces challenges such as poor angular resolution [9], sparse point clouds [10], severe interference and noise [11], and poor interpretation ability [12], which limit its widespread adoption.

This paper addresses the challenges faced by MIMO millimeter-wave radar in environmental reconstruction, such as poor angular resolution, significant interference and noise, and sparse point clouds. We propose a method for high-resolution 3D environmental map construction using millimeter-wave radar. The main contributions of the paper are briefly summarized as follows.

- A method for constructing three-dimensional environmental maps using millimeter-wave radar aimed at scene reconstruction is proposed, generating high-density three-dimensional environmental point clouds comparable to those of multi-line LiDAR.
- A framework for semantic SLAM using millimeter-wave radar is proposed. On the one hand, it optimizes the point cloud density of SLAM environmental maps through semantic information learning. On the other hand, SLAM assists in enhancing the effectiveness of semantic deep learning training.
- Experiments across various scenarios were conducted to verify the effectiveness of the proposed methods in improving resolution, increasing point cloud density, and suppressing noise and interference.

The rest of this paper is structured as follows. Section 2 gives a brief overview of related works on high-resolution millimeter-wave radar environmental mapping tasks. Section 3 introduces the proposed high-resolution millimeter-wave radar 3D mapping method. Section 4 describes the experimental results and performance analysis. Finally, Section 5 concludes this paper.

## 2. Related Work

Improving the effectiveness of millimeter-wave radar environmental map construction mainly involves two technical approaches: traditional signal processing methods and deep learning methods.

For traditional signal processing methods, the essence lies in enhancing the radar angular resolution by increasing the radar aperture, which, combined with radar SLAM algorithms, achieves high-resolution radar environmental mapping. Increasing the radar aperture can be achieved through techniques such as array radar virtual aperture, synthetic aperture, and multi-view observation fusion. Array radar virtual aperture technology arranges multiple transmitting and receiving antennas spatially to form MIMO array radar. MIMO array radar simultaneously transmits and receives multiple beams, which can focus on different azimuth and elevation angles independently. By combining and processing these beams, higher resolution and more precise target information can be obtained, thereby improving radar imaging quality [13,14]. Super-resolution imaging technology is often used to further enhance the resolution of MIMO array radar. By exploiting the sparsity assumption of radar imaging, super-resolution imaging technology breaks through the physical aperture limitation of radar, achieving over 5 times enhancement in radar imaging resolution. Although increasing the number of antennas continuously enlarges the aperture of a single radar and can improve the angular resolution, the time-division orthogonal transmission in MIMO systems reduces the radar’s velocity measurement range [15]. The coordinated observation of targets by multiple spatially distributed small-aperture radars is a method for increasing the radar’s physical aperture at the data processing level. Distributed radars form a large-aperture sparse array in space, thus achieving high-resolution environmental reconstruction of specific areas [16,17]. Compared to increasing the radar aperture in the spatial domain, synthetic aperture technology embodies the idea of trading time for space. A single small-aperture radar moving in space can also form a virtual aperture array. This method of increasing the radar spatial domain through radar motion in the time domain offers higher degrees of freedom compared to increasing the radar aperture in the spatial domain, resulting in higher-resolution environmental reconstruction [18,19]. However, synthetic aperture technology requires the observed scene to remain stationary during the synthetic aperture time and precise trajectory and pose information for motion compensation.

Based on deep learning, the method for constructing high-resolution radar environmental maps differs from traditional signal processing approaches in that it does not involve a specific radar mode design. Instead, it leverages other high-resolution sensors, such as LiDAR, cameras, etc., to train millimeter-wave radar in a data-driven manner to enhance imaging quality. This enables millimeter-wave radar to handle high-resolution environmental mapping tasks. Early work primarily focused on reconstructing specific nearby targets [20,21,22,23], using 3D point clouds obtained from depth cameras as the ground truth to train deep learning networks to generate high-density radar point clouds of specific targets, like vehicles. In environmental mapping research targeting scenes, 2D LiDAR scan maps were primarily used as the ground truth, constructing convolutional neural networks to train millimeter-wave radar distance–azimuth heatmaps, enabling millimeter-wave radar to achieve 2D environmental reconstruction comparable to LiDAR results [24,25,26]. In recent years, building on 2D scene reconstruction, efforts have been made toward indoor environment reconstruction [27], utilizing deep learning to generate dense indoor point clouds for training millimeter-wave radar point clouds, thereby enabling millimeter-wave radar to reconstruct three-dimensional indoor environments for small-scale scenes. The deep learning-based method for constructing high-resolution radar environmental maps achieves higher accuracy and robustness with reduced radar system complexity compared to traditional signal processing methods. However, radar datasets are significantly more challenging and less stable to acquire compared to visual datasets, leading to the generally poorer robustness of deep learning methods applied to radar data.

Regardless of whether traditional signal processing methods or deep learning approaches are used, current research on high-resolution radar environmental mapping faces several challenges.

- In terms of algorithm practicality, existing methods are predominantly limited to reconstructing three-dimensional representations of specific targets and small indoor scenes, failing to meet the demands of larger outdoor environments.
- In terms of algorithm robustness, current methods struggle to adapt to the dynamic requirements of environmental mapping in real-time scenarios. Present deep learning networks typically operate with two types of inputs: one based on radar heatmaps, which suffer from sparsity and often fail to capture all details of a scene using a single heatmap, and the other based on sequences of radar heatmaps, which necessitate slow and consistent radar platform movement, thus precluding the accommodation of non-uniform platform motion.
- In terms of algorithm scalability, traditional signal processing methods and deep learning approaches have predominantly been pursued independently in most studies. Traditional methods rely on engineering expertise for design, whereas deep learning methods heavily rely on extensive datasets for performance enhancement. Each approach has its strengths, and combining the two could potentially yield superior performance and effectiveness.

Addressing the challenges faced in current high-resolution radar 3D environmental mapping and the application demands for large outdoor scenes, this paper proposes a high-resolution millimeter-wave radar 3D environmental reconstruction method that integrates radar signal processing SLAM algorithms with radar deep learning techniques.

## 3. Methodology

### 3.1. Method Pipeline

**Figure 1:**
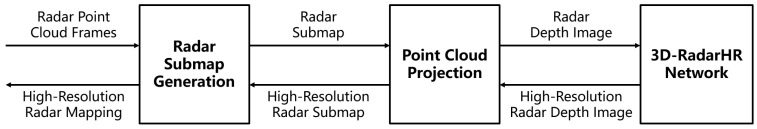
The flowchart of the method for constructing high-resolution 3D environmental maps using millimeter-wave radar.

This paper presents a method for high-resolution 3D environmental mapping using millimeter-wave radar, which embodies the concept of semantic SLAM. The method first utilizes SLAM to assist in semantic perception and then integrates the semantic results into SLAM to construct more detailed environmental maps. The flowchart of the method is illustrated in Figure 1, which primarily includes three parts: radar submap generation, point cloud projection, and the 3D-RadarHR network.

Millimeter-wave radar echo signals processed through radar signal processing yield three-dimensional spatial $\left(x,y,z\right)$, velocity $\left(v\right)$, RCS, and other information, forming radar point clouds [3,20,26]. Due to radar resolution limitations, millimeter-wave radar point clouds generally exhibit densities over 100 times lower than those of LiDAR point clouds. The sparsity of radar point clouds severely compromises the robustness of deep learning tasks based on single-frame radar point clouds. Consequently, numerous studies [25,26,27,28] have utilized radar point cloud temporal sequences as input data for deep learning networks. However, within radar point cloud sequences, the temporal and spatial variations in radar point cloud frames are considered relatively constant. Challenges arise in applications involving platforms with variable speeds, such as autonomous driving, which can destabilize model consistency.

This paper proposes integrating advanced radar SLAM to transform traditional methods of radar point cloud temporal sequences into submaps. This approach enables adaptation to applications involving radar platform motion with variable speeds, enhancing the purity of input data for backend deep learning methods. Sensors like LiDAR and depth cameras can capture high-quality environmental point clouds and are often used as teacher sensors to train millimeter-wave radar to acquire high-density point clouds. Optical depth cameras are primarily used for indoor reconstruction, typically effective within distances not exceeding 10 m, and are thus unsuitable for outdoor environmental reconstruction. Consequently, this paper selects LiDAR as the teacher sensor.

The operational frequency of millimeter-wave radar is 1000 times lower than that of LiDAR, resulting in two main disparities between millimeter-wave radar and LiDAR point clouds. Firstly, millimeter-wave radar exhibits a lower point cloud density due to poorer resolution. Secondly, millimeter-wave radar involves complex electromagnetic wave diffraction mechanisms, allowing it to detect partially occluded objects. To enhance the robustness of deep learning methods and stabilize the resulting millimeter-wave radar point clouds, this paper proposes a normalization process for the point clouds obtained from both sensors before deep learning. This normalization involves point cloud projection, with two primary objectives: appropriately reducing LiDAR resolution and eliminating point clouds formed by millimeter-wave radar due to electromagnetic wave diffraction. Following data preprocessing, the 3D-RadarHR network reconstructs high-resolution millimeter-wave radar point clouds, combining radar SLAM methods to ultimately construct high-resolution environmental maps.

### 3.2. Radar Submap Generation

This paper proposes to enhance the robustness of the system by utilizing advanced radar SLAM methods to generate radar submaps from multiple frames of millimeter-wave radar point clouds as input data for deep learning, replacing traditional radar point cloud time-series approaches. This adaptation enables the method proposed in this paper to handle the task of environmental map construction under the varying-speed motion of radar platforms.

The radar submap construction proposed in this paper primarily employs the radar Doppler odometry algorithm. The core of this method is to regressively analyze the angle and velocity information within radar point clouds to estimate the forward and rotational speeds of the vehicle. Subsequently, the accurate estimation of the vehicle pose is achieved through trajectory accumulation. The Doppler odometry algorithm is well suited for short-distance radar SLAM tasks. However, due to the absence of backend optimizations such as loop-closure detection, it exhibits significant error accumulation issues in large-scale SLAM tasks.

**Figure 2:**
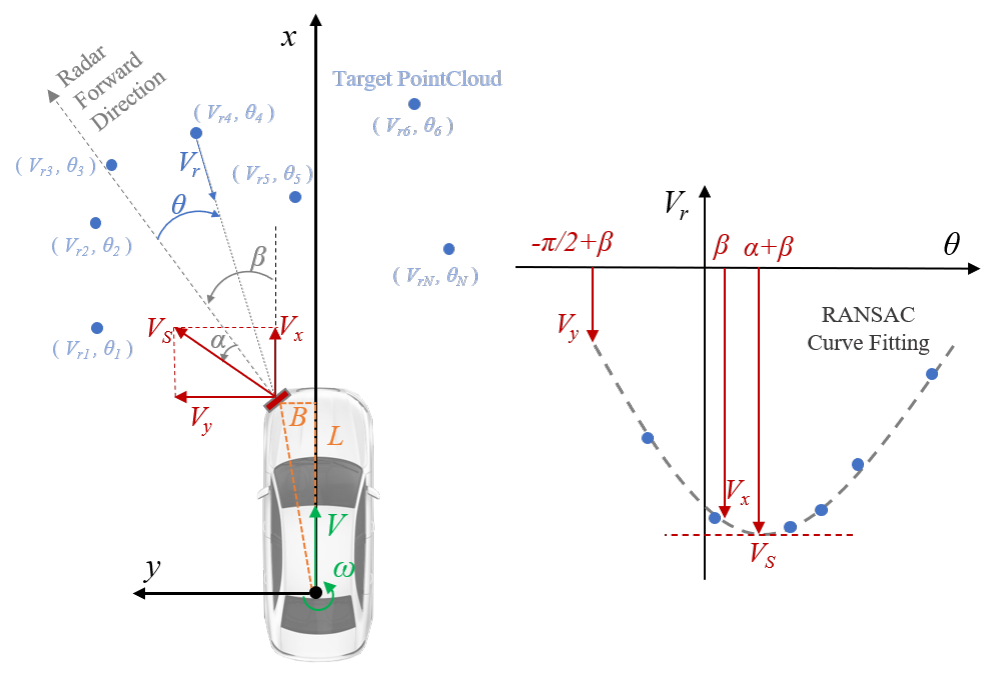
The principle of millimeter-wave radar Doppler odometry. The left diagram illustrates the physical relationships among the variables. The right diagram depicts the mathematical relationships among the variables.

Figure 2 illustrates the principle of radar odometry based on Doppler information. Radar odometry based on Doppler information mainly requires the use of information on the azimuthal angle $\theta_{n}$ and radial velocity $V_{rn}$ in the N-point radar point cloud. For the point set extracted by the radar, the cosine curve can be fitted by the random sample consensus (RANSAC) algorithm. Then, the forward velocity *V* and the rotational angular velocity ω of the vehicle can be solved using the parameters of the fitted cosine curve.

First, the point set $\left(\theta_{n},V_{rn}\right)$ is fitted with a cosine curve using the RANSAC algorithm. Then, the radar’s velocity $V_{s}$, and yaw angle α can be estimated from the vertices of the resulting cosine curve. When the yaw angle of the radar is β, the radar velocity $V_{s}$ is decomposed along the direction of the x-axis (forward) and y-axis (left), and the velocity component of the radar velocity along the front and side can be obtained

(1) $$\left\{\begin{array}{c} V_{x}=V_{s}\times \cos\left(\alpha +\beta \right)\\ V_{y}=V_{s}\times \sin\left(\alpha +\beta \right) \end{array}\right.$$

By combining the $\left(V_{x},V_{y}\right)$ obtained with the location of the radar mount, the following relation is satisfied when the vehicle satisfies Ackerman’s assumption

(2) $$\left\{\begin{array}{c} V_{x}=V-\omega \cdot B\\ V_{y}=\omega \cdot L \end{array}\right.$$

where *L* is the offset between the position of the radar mount and the center of rotation of the vehicle in the forward direction of the vehicle, and *B* is the offset between the position of the radar mount and the center of rotation of the vehicle in the left direction of the vehicle.

The vehicle velocity can be calculated as follows.

(3) $$\left\{\begin{array}{c} \omega =\frac{V_{y}}{L}=\frac{V_{s}\sin\left(\alpha +\beta \right)}{L}\\ V=V_{x}+\omega B=V_{s}\cos\left(\alpha +\beta \right)+\frac{B}{L}V_{s}\sin\left(\alpha +\beta \right) \end{array}\right.$$

The performance of vehicle speed estimation using radar odometry is analyzed in reference [15].

**Figure 3:**
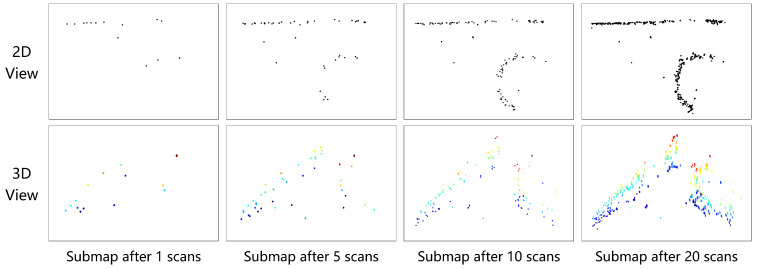
Comparison of radar submap construction results with different numbers of frames in subterranean tunnel intersection scenes.

Figure 3 illustrates the environmental map construction results obtained by building radar submaps from 1, 5, 10, and 20 frames of point clouds in subterranean tunnel scenarios. As shown in Figure 3, a single frame of the radar point cloud is extremely sparse, and structural information on the tunnel walls and other environmental features cannot be effectively displayed. With an increase in the number of radar point cloud frames, environmental structural information becomes progressively clearer. When the number of frames used for radar submap construction reaches 20, both the 2D and 3D point clouds achieve a high point cloud density, visually presenting environmental information effectively in the constructed submaps.

### 3.3. Depth Image Representation of Point Clouds

After the millimeter-wave radar point cloud is processed by radar odometry to construct submaps, the point cloud density is greatly enhanced. However, compared to the environmental point cloud data acquired by LiDAR, there exist two main differences. The first difference lies in the point cloud density: the current state-of-the-art 4D millimeter-wave radar achieves an angular resolution of up to 1°, whereas LiDAR typically exceeds 0.1° in angular resolution. Although radar odometry accumulates multiple frames of radar point clouds over multiple instances to achieve the densification of millimeter-wave radar point clouds, limitations in resolution mean that millimeter-wave radar still lags behind optical sensors such as LiDAR in representing the fine structural details of the scene. The second difference pertains to point cloud attributes: millimeter-wave radar operates in the millimeter-wave band, while optical sensors operate in the sub-micrometer band. The longer wavelength of millimeter-wave radar endows it with stronger diffraction properties compared to LiDAR. Diffraction allows millimeter-wave radar to effectively detect partially obscured objects, thereby enabling the detection of non-line-of-sight targets that optical sensors might miss. This characteristic potentially enriches the millimeter-wave radar point cloud with objects that are undetectable by optical sensors. The aforementioned differences reduce the point cloud correspondence between LiDAR and millimeter-wave radar. To ensure high computational efficiency, the network architecture must remain relatively simple. Therefore, to effectively train millimeter-wave radar using LiDAR data within a simplified network structure, it is essential to address these discrepancies arising from their distinct imaging mechanisms. Otherwise, the trained millimeter-wave radar will lack generalizability and robustness.

**Figure 4:**
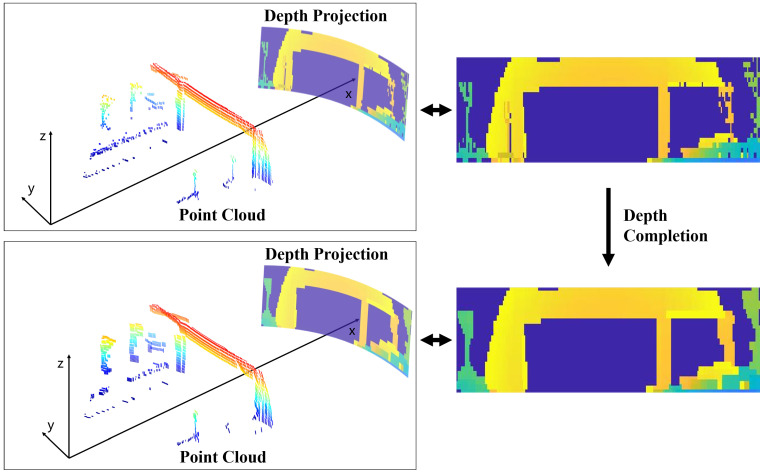
An illustration of the effectiveness of millimeter-wave radar point cloud depth image projection.

To tackle these issues, this paper proposes a normalization process between millimeter-wave radar point cloud data and LiDAR point cloud data before constructing deep learning models. This normalization process, facilitated by point cloud projection algorithms, serves two primary purposes: appropriately reducing LiDAR resolution and eliminating additional point clouds formed by diffraction phenomena in millimeter-wave radar. A schematic diagram of the millimeter-wave radar point cloud depth image projection algorithm is shown in Figure 4. The 3D point clouds from millimeter-wave radar or LiDAR in three-dimensional space are first projected from the Cartesian coordinate system, $\left(r,\theta ,z\right)=(\sqrt{x^{2}+y^{2}},arctan\left(y/x\right),z)$. Subsequently, the 3D point cloud is projected along the depth dimension r onto a 2D cylindrical surface, where multiple targets from the same angle but different distances are reduced to only the nearest one, thereby eliminating non-line-of-sight targets in the millimeter-wave radar point cloud. The point cloud is then discretized into a depth image using a specified grid size, which may reduce the resolution of the LiDAR point cloud to some extent. However, projecting the 3D point cloud into a depth image introduces holes in the image, affecting pixel continuity. Therefore, the depth image needs to be completed by filling these holes. For a sparse depth image $D_{sparse}\in R^{M\times N}$ with holes, depth image completion requires finding a transformation function $f\left(\cdot \right)$, sometimes assisted by a guidance image $I\in R^{M\times N}$, such that $f\left(I,D_{sparse}\right)$ approaches the hole-free true depth image $D_{dense}\in R^{M\times N}$, which solves the following optimization problem.

(4) $$\min_{f}{∥f\left(I,D_{sparse}\right)-D_{dense}∥}_{F}^{2}$$

This paper employs a method based on digital image morphology for depth image completion [29]. This approach can operate independently without requiring a guidance image while also potentially blurring details in the depth image, further reducing the resolution of the LiDAR. By applying identical parameter settings for point cloud projection onto both LiDAR point clouds and submap point clouds from millimeter-wave radar environments, radar and depth images with similar resolutions can be obtained.

### 3.4. The 3D-RadarHR Network

After constructing submaps using millimeter-wave radar and aligning LiDAR and millimeter-wave radar point clouds, the main difference between the LiDAR and millimeter-wave radar lies only in resolution. This paper proposes a deep learning method for generating high-resolution 3D point clouds from millimeter-wave radar, termed 3D-RadarHR networks. Utilizing a U-shaped sparse convolutional network structure, 3D-RadarHR networks take depth images formed from millimeter-wave radar submaps as input and aim to produce high-resolution millimeter-wave radar depth images similar to those from LiDAR through training.

Different from traditional data formats based on radar image sequences, radar point clouds, or radar 3D spatial grids, the data format used in this paper is more compact, suitable for applications such as SLAM that require real-time performance. This paper investigates the performance metrics of common 4D millimeter-wave radars. Typically, the azimuthal 3 dB beamwidth is 80° with an azimuthal angular resolution of 2°, while the elevational 3 dB beamwidth is 40° with an elevational angular resolution of 4°. In contrast, high-performance multi-line LiDAR scanners offer a 360° azimuthal scanning range with an azimuthal angular resolution of 0.1° and a 40° elevational scanning range with an elevational angular resolution of 0.2°. Therefore, this paper selects an azimuthal angle range of −40 to 40° and an elevational angle range of −20 to 20° in the beam overlap region of the two sensors. The reconstructed millimeter-wave radar has an azimuthal angular resolution of 0.25° and an elevational angular resolution of 0.5°, resulting in input and output depth map sizes of 320 × 80 for 3D-RadarHR Networks.

**Figure 5:**
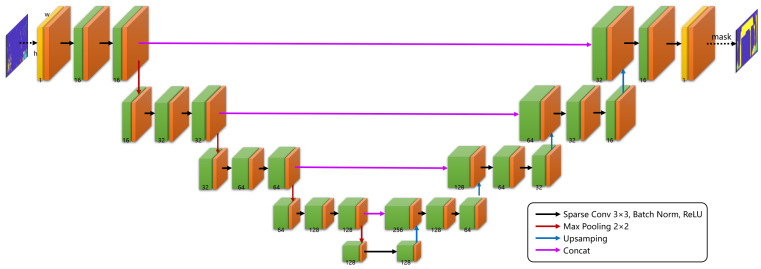
A diagram of the 3D-RadarHR deep learning network architecture.

The 3D-RadarHR network architecture is depicted in Figure 5. For the depth images obtained by projecting radar submaps, the first step involves binarization to obtain the observation mask layer *o*, corresponding to the orange part in Figure 5. In *o*, regions where the value is 1 indicate observed values in the input image, while regions with a value of 0 indicate no observations at those locations. Sparse convolution operations can be defined based on the observation mask. Traditional convolution operations are outlined as follows.

(5) $$f_{u,v}\left(x\right)=\sum_{i,j=-k}^{k}x_{u+i,v+j}\cdot w_{i,j}+b$$

where *x* represents the input image, *w* denotes the convolution kernel weights, *b* is the bias term, *u* and *v* denote the pixel coordinates of the input image, and *k* indicates the kernel size. For sparse data, a binary mask called the observation mask can be used during the convolution process to indicate missing elements, ensuring that the network’s performance is not affected by the sparsity of input data [30]. The sparse convolution operation is defined as follows.

(6) $$f_{u,v}\left(x,o\right)=\frac{\sum_{i,j=-k}^{k}o_{u+i,v+j}\cdot x_{u+i,v+j}\cdot w_{i,j}}{\sum_{i,j=-k}^{k}o_{u+i,v+j}+\varepsilon }+b$$

In this case, *o* represents the observation mask, and ε is a constant used to avoid division by zero.

After passing through a series of sparse convolution layers, Batch Normalization layers, and ReLU activation layers, the input image undergoes downsampling via max pooling to capture semantic understanding, thereby functioning as an encoder. After four layers of downsampling, the feature image is then upsampled using nearest-neighbor interpolation to restore high-resolution images, functioning as a decoder. Additionally, in the multi-layer deep network, in order to enhance network robustness and preserve the low-dimensional features of the image, connections are established between the encoder and decoder at the same scale, forming a structure similar to residual networks (ResNet).

To measure the disparity between the expected output and the actual output of the network, the loss function employed in this paper is as follows.

(7) $$\begin{array}{cc} Loss & =\alpha DiceLoss+\left(1-\alpha \right)BCELoss\\ \; & =\alpha \left(1-\frac{2\sum_{i=1}^{N}y_{i}g_{i}}{\sum_{i=1}^{N}y_{i}^{2}+\sum_{i=1}^{N}g_{i}^{2}}\right)+\left(1-\alpha \right)\left[\frac{1}{N}\sum_{i=1}^{N}y_{i}\log g_{i}+\left(1-y_{i}\right)\log\left(1-g_{i}\right)\right] \end{array}$$

where $y_{i}$ represents the actual output of the network, $g_{i}$ denotes the expected output, *N* stands for the number of image pixels, and α is a weighting coefficient. This loss function combines DiceLoss and BCELoss with a weighted approach. The DiceLoss considers the overlap between the actual and expected output images, enhancing the network’s sensitivity to edge pixels. The BCELoss independently calculates pixel-wise differences between the actual and expected outputs, thereby improving the overall accuracy of pixel reconstruction by the network. In our study, through testing, the 3D-RadarHR network exhibited optimal performance when α=0.3.

## 4. Experiments and Analysis

### 4.1. Experimental Setup

**Figure 6:**
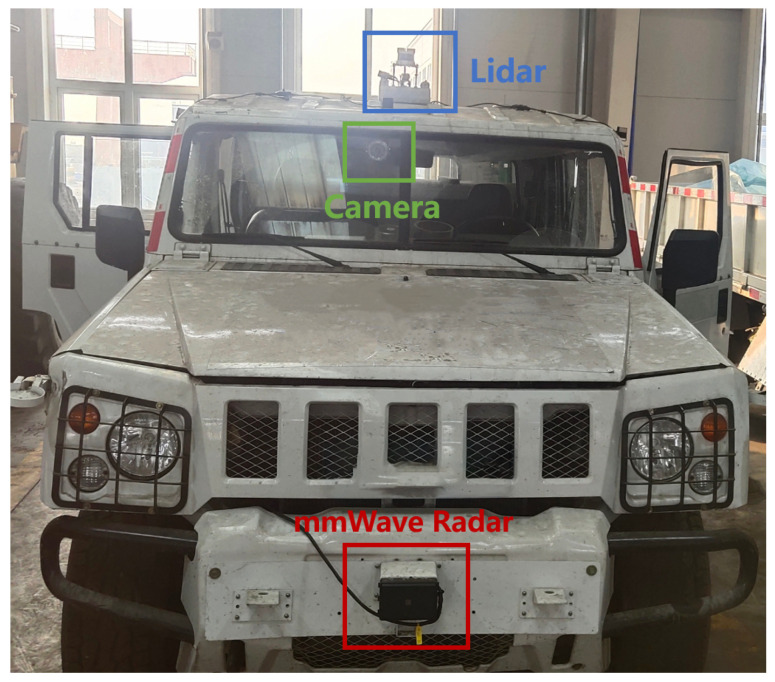
A vehicle equipped with millimeter-wave radar, LiDAR, and a camera.

All experiments were carried out using a pickup truck, as shown in Figure 6, where the camera, LiDAR, and millimeter-wave radar sensors were logged via the vehicle’s Controller Area Network (CAN) bus. A 77–79 GHz millimeter-wave radar mounted on the front of the vehicle features 12 transmitting antennas and 16 receiving antennas, with an azimuthal field of view of ±60° and an azimuthal angular resolution of 2°. The elevation field of view is ±20° with an elevation angular resolution of 3°. A 128-line LiDAR is installed on the vehicle roof, providing reference values for millimeter-wave radar environmental perception results. The vehicle is also equipped with GNSS and an IMU to provide high-precision attitude information and vehicle position in open outdoor scenarios. The millimeter-wave radar, LiDAR, and cameras synchronize data acquisition through software at a frame rate of 10 Hz. The experimental environments included outdoor and underground scenarios, and approximately 126,180 samples of valid data were collected, with outdoor environment data accounting for 40% and subterranean environment data for 60%.

The radar signal processing algorithm was developed based on Python. The whole system runs on a computer with a CPU (i7-8750H) at 2.2 GHz and a memory of 16 GB. And, we implemented the deep learning model based on Pytorch running on an NVIDA Quadro RTX 4000 GPU.

### 4.2. Experimental Results

**Figure 7:**
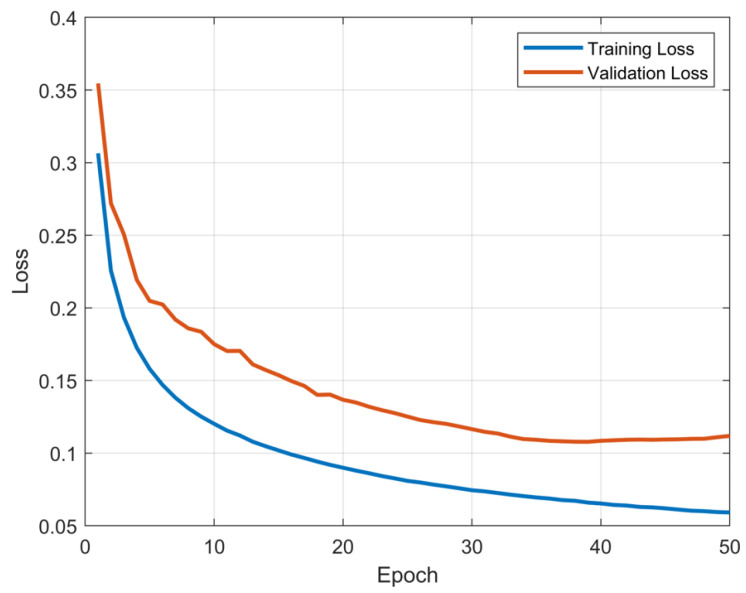
The training and validation loss curves for 3D-RadarHR.

A dataset was constructed based on experimental data collected from sensors, including millimeter-wave radar, LiDAR, camera, IMUs, etc., amounting to a total of 126,180 valid data samples. We acquired about 75,708 samples of training data and about 25,236 samples of validation/development data to be used for training the model. About 25,236 samples of test data were also collected with a human subject. The model was trained using the Adam optimizer with the objective of minimizing Equation (7) of the output with respect to the ground-truth data. The training and validation loss curves for 3D-RadarHR are shown in Figure 7.

**Figure 8:**
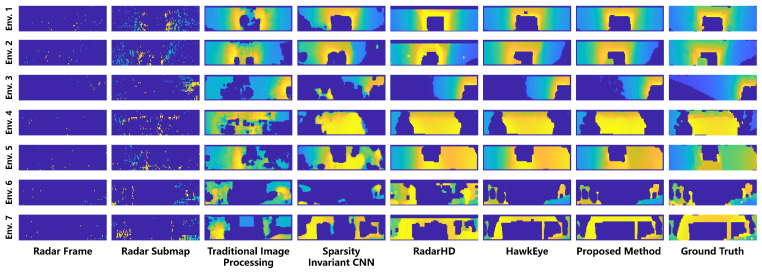
Comparison of millimeter-wave radar depth image reconstruction results using Radar Frame, Radar Submap, Traditional Image Processing [29], Sparsity Invariant CNN [30], RadarHD [20], HawkEye [20], and Proposed Method.

The 3D-RadarHR network was evaluated using our dataset. Figure 8 compares the scene depth images obtained by different methods across seven typical scenarios. Each row in the figure represents a different scenario. Env. 1 and Env. 2 depict straight underground mine passages, Env. 3 shows a curved underground mine passage, Env. 4 represents a wall scene, Env. 5 shows an intersection in a subterranean passage, Env. 6 depicts an outdoor road scene, and Env. 7 shows an outdoor memorial archway scene. Each column in the figure represents depth images obtained by different methods: the first column shows single-frame radar depth images, the second column shows radar submap depth images, and columns three through seven show results based on further processing of radar submap depth images. Specifically, column three displays results from traditional image processing methods for radar depth image completion [29], column four shows results from Sparsity-Invariant CNN [30] for radar depth image reconstruction, column five shows results from the RadarHD network [26] for radar depth image reconstruction, column six shows results from the HawkEye network [20] for radar depth image reconstruction, column seven displays results from our proposed method for radar depth image reconstruction, and column eight shows the reference ground truth obtained by completing and downsampling depth images from a 128-line LiDAR depth image. Based on Figure 8, a qualitative assessment of the reconstruction results from various methods can be conducted. The radar depth images obtained after radar submap construction show a significant improvement in quality compared to single-frame radar images, allowing for the observation of approximate scene contour information.

To quantitatively evaluate the reconstruction effectiveness of different methods, the Mean Absolute Error (MAE) and Root Mean Square Error (RMSE) are adopted as evaluation metrics [31]. The MAE and RMSE are, respectively, used to assess the first and second moments of errors, reflecting the mean and variance of image reconstruction quality.

(8) $$MAE=\frac{1}{N}\sum_{i=1}^{N}\left|y_{i}-g_{i}\right|$$

(9) $$RMSE=\sqrt{\frac{1}{N}\sum_{i=1}^{N}{\left(y_{i}-g_{i}\right)}^{2}}$$

Table 1 and Table 2 present the quantitative evaluation results of MAE and RMSE for various methods across the typical scenarios depicted in Figure 8. Table 3 displays the average computational frame rates of these methods. In terms of radar depth image reconstruction quality, both our proposed method and the HawkEye approach significantly outperform other algorithms, achieving notably good reconstruction results. Regarding algorithm efficiency, our method demonstrates higher execution efficiency. Specifically, for a 10 Hz millimeter-wave radar sensor, it meets real-time processing requirements. This is attributed to our approach employing radar SLAM techniques to generate 2D submaps from multi-frame radar depth images before feeding them into the neural network. This preprocessing step reduces the network’s parameter size substantially compared to the HawkEye approach, which uses a larger-scale, multi-frame 3D data input, thereby increasing computational complexity. By integrating signal processing and information processing effectively, our method not only ensures high-quality reconstructed images but also greatly enhances operational efficiency.

**Figure 9:**
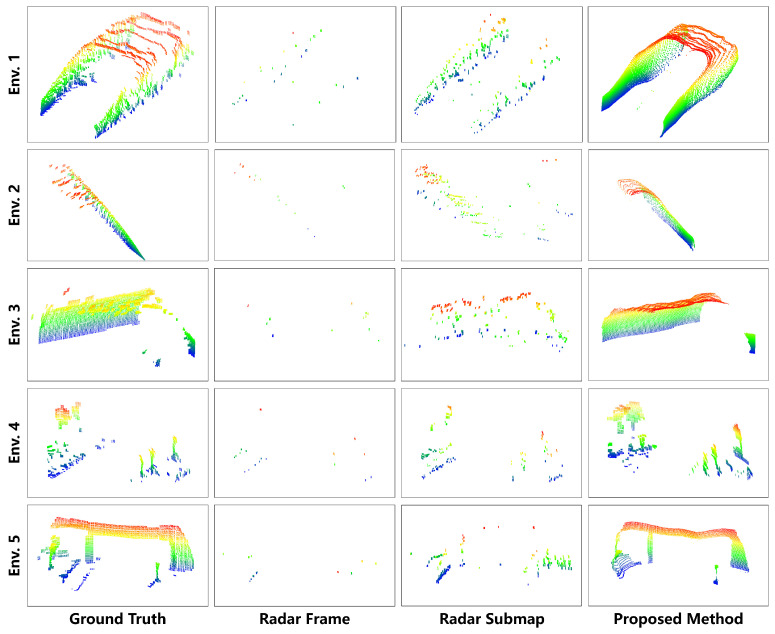
High-resolution 3D point cloud reconstruction results using millimeter-wave radar.

After achieving the high-resolution reconstruction of radar depth images, the transformed relationship between depth images and 3D point clouds, as shown in Figure 8, yields the reconstructed radar-resolution 3D point clouds depicted in Figure 9. Each row in the figure represents different scenes, while each column represents different methods. It is evident that our method significantly enhances the point cloud density and resolution compared to the original radar point clouds, approaching the quality of high-resolution LiDAR. Furthermore, our method demonstrates the capability to filter out noise and interference from the original millimeter-wave radar point clouds, enhancing detail preservation. Specifically, the noise suppression capability of the proposed method stems from two main aspects. First, the radar submap construction algorithm, as illustrated in Figure 2, plays a crucial role; the point cloud of static targets tends to be distributed along a cosine curve in the azimuth and radial velocity dimensions. In contrast, point clouds resulting from moving target interference, the side-lobe interference of static targets, and random noise do not conform to this constraint. Therefore, by employing a cosine-curve-fitting algorithm to construct the radar submap, the method effectively filters out noise. Second, the 3D-RadarHR deep learning network uses LiDAR point clouds as the target output. Compared to millimeter-wave radar, LiDAR offers higher spatial resolution and measurement accuracy while being less susceptible to multipath scattering and background noise. These characteristics contribute to an overall improvement in the SNR of the millimeter-wave radar point clouds.

### 4.3. Performance Analysis

This section analyzes the performance advantages of the proposed method over existing approaches in scenarios involving variable-speed motion. Additionally, it examines the impact of the Density Growth Factor of the Radar Submap on the quality of radar depth image reconstruction by the 3D-RadarHR network under the condition of low radar platform motion error.

#### 4.3.1. Performance Comparison in Variable-Speed Motion Applications

**Figure 10:**
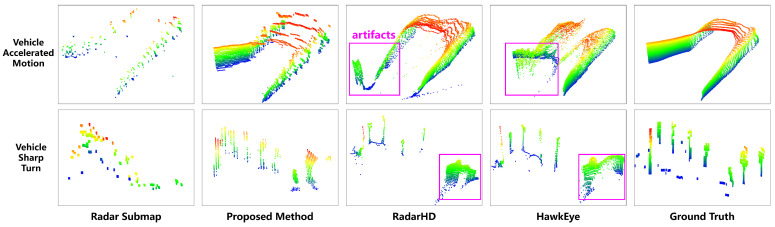
Comparison of 3D mapping using Radar Submap, Proposed Method, RadarHD [26], HawkEye [20] under sudden changes in vehicle speed. The purple bounding box area highlights the artifact regions in the map reconstruction.

This section compares the 3D environmental map construction performance of different methods when the vehicle undergoes sudden changes in speed, highlighting the contribution of the submap construction module in the proposed method, as shown in Figure 10. Two specific scenarios are included in the comparison. In the first scenario, the vehicle moves in a straight line with varying speeds, with the experimental setting being a mine tunnel intersection. In this case, existing methods, such as RadarHD and HawkEye, which directly use point cloud frame sequences as input, exhibit blurred reconstructions of the left-side intersection. The second scenario involves a sharp vehicle turn, with the experimental setting being an outdoor intersection. Here, the existing methods suffer from point cloud smearing due to rotational effects. However, in both of these typical scenarios, the proposed method demonstrates significantly superior environmental point cloud map construction. Existing methods typically use point cloud frame sequences as input. However, due to the inherent sparsity of millimeter-wave radar point clouds, it is challenging for deep learning networks to directly learn the transformation relationships between poses of consecutive frames. As a result, deep learning networks often assume that these point cloud sequences exhibit approximately uniform linear motion. In this work, by incorporating Doppler information from millimeter-wave radar in the submap construction preprocessing stage, we reduce the pose uncertainty errors associated with directly using point cloud frame sequences for deep learning. This enables the proposed method to perform favorably in scenarios involving non-uniform motion. Based on the above analysis, if one seeks to continue using point cloud frame sequences as input to accurately reconstruct environmental maps, two improvements are recommended: first, incorporating Doppler information and using four-dimensional millimeter-wave radar point clouds (three spatial dimensions and Doppler information) as input; second, designing a dedicated radar point cloud pose estimation network and collecting a sufficiently large dataset that reflects non-uniform motion. Nevertheless, both approaches would increase the computational and memory demands of the deep learning network, which warrants further investigation in future research.

#### 4.3.2. Impact of Radar Submap on 3D-RadarHR Network Reconstruction Errors

**Figure 11:**
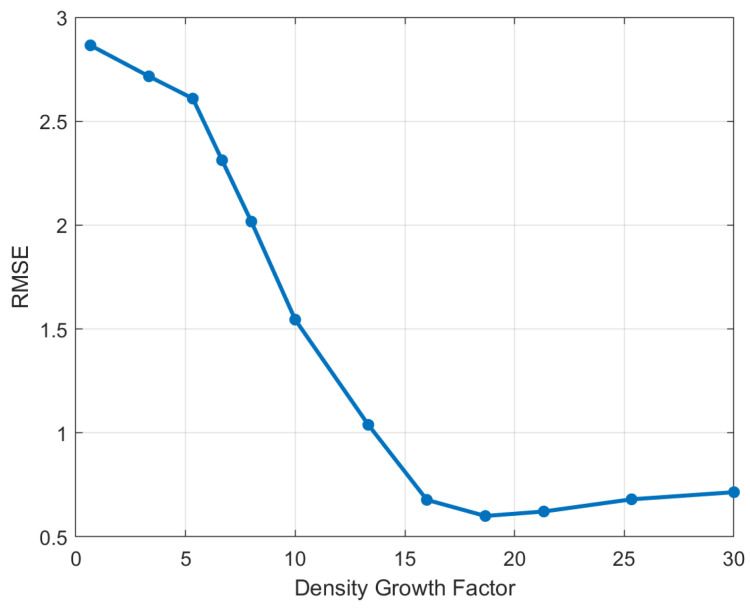
The impact of the Density Growth Factor on the 3D-RadarHR network error in radar submap construction.

To quantify the point cloud density increment provided by the radar submap relative to single radar point cloud frames, we define the ratio of non-zero pixels in the radar submap depth image to those in the radar frame depth image as the Density Growth Factor of the Radar Submap. This metric is more effective than simply counting the cumulative number of frames in the submap, as it avoids issues of redundant frame data when the vehicle is moving very slowly or stationary. Figure 11 illustrates the impact of the Density Growth Factor on the error rate of the 3D-RadarHR network. In the initial stages, as the Density Growth Factor increases, the reconstructed environmental map exhibits higher point cloud density and accuracy. However, when the Density Growth Factor reaches a certain threshold, around 18 in this case, the contribution of further submap construction to the 3D-RadarHR network’s performance levels off. Beyond this point, further increasing the Density Growth Factor leads to a decline in network performance. The primary cause of this trend is the accumulated error in the odometry calculation used for radar submap construction. While replacing the current odometry method with a more precise one could improve network accuracy, accumulating too many frames increases both data acquisition and processing times, thereby reducing real-time performance. Therefore, the choice of the number of frames in the radar submap must balance accuracy and complexity. In this paper, we set the Density Growth Factor to 18.

### 4.4. Mapping Comparison

This section will present comprehensive experiments conducted on environmental map construction with the millimeter-wave radar SLAM and semantic environmental mapping algorithms proposed in this paper, and analyze the experimental results.

**Figure 12:**
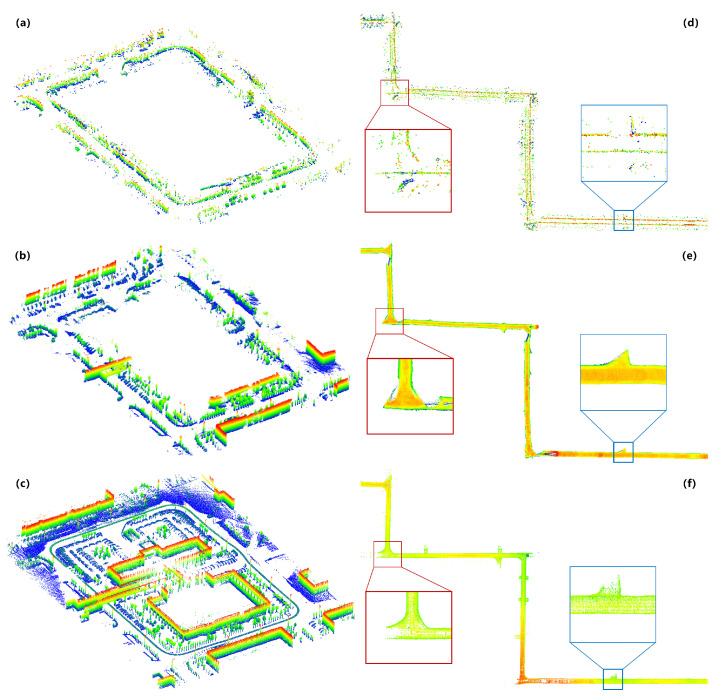
Comparison of environmental map construction results (**a**) Direct mapping result of millimeter-wave radar SLAM for outdoor scene (**b**) Semantic mapping result of millimeter-wave radar SLAM for outdoor scene (**c**) Mapping result of LiDAR SLAM for outdoor scene (**d**) Direct mapping result of millimeter-wave radar SLAM for subterranean tunnel scene (**e**) Semantic mapping result of millimeter-wave radar SLAM for subterranean tunnel scene (**f**) Mapping result of LiDAR SLAM for subterranean tunnel scene.

Figure 12a–c compare the outdoor SLAM environmental map construction results. In comparison, Figure 12a shows the direct mapping result using millimeter-wave radar SLAM, while Figure 12c shows the direct mapping result using LiDAR SLAM. Although the millimeter-wave radar SLAM algorithm proposed in this paper provides accurate localization results, the three-dimensional point cloud map still exhibits considerable noise and sparse points, especially in the pitch dimension. Additionally, due to the limited angular resolution of millimeter-wave radar, only point clouds within 20 m were used to construct the environmental map as densely as possible. Overall, the radar point cloud map at this stage only serves the function of distinguishing occupied and free areas, with relatively weak semantic information. Figure 12b displays the three-dimensional dense point cloud map obtained by integrating the millimeter-wave radar SLAM with the semantic environmental mapping algorithm proposed in this paper. Compared to the direct mapping results of millimeter-wave radar, the environmental map at this stage shows two significant improvements. Firstly, the point cloud density is higher, allowing the clear differentiation of architectural walls, trees, and other objects, thereby significantly enhancing semantic information. Secondly, noise in the point cloud has been effectively suppressed, achieving mapping results comparable to those of multi-line LiDAR.

Figure 12d–f illustrate the comparison of the SLAM environmental map construction results in subterranean tunnel scenes. Figure 12d shows the direct mapping result using millimeter-wave radar SLAM, while Figure 12e presents the mapping result using the millimeter-wave radar SLAM algorithm proposed in this paper along with semantic environmental map construction. Figure 12f depicts the mapping result using multi-line LiDAR. Similar to outdoor scene experiments, our method in this study achieves dense environmental point cloud maps comparable to LiDAR. Additionally, our method exhibits multipath suppression capability. Specifically, as shown in the red-boxed area of the figure, direct mapping results from millimeter-wave radar SLAM exhibit multipath interference at an L-shaped intersection, whereas semantic mapping results effectively filter out this interference. This multipath filtering capability, distinct from the non-line-of-sight environmental map construction method proposed in this paper, requires no prior environmental information. The multipath filtering capability primarily derives from two aspects: first, depth image projection, which retains only the nearest radar point clouds from the same perspective, and second, supervised deep learning training using LiDAR without multipath interference.

## 5. Conclusions

This paper presents a high-resolution 3D environmental mapping algorithm for millimeter-wave radar. The algorithm integrates methods for processing odometry signals in millimeter-wave radar SLAM with deep learning information processing. By utilizing millimeter-wave radar odometry-assisted deep learning training, the deep learning framework provides feedback for millimeter-wave radar SLAM environmental map construction, resulting in dense environmental point cloud maps. Experimental results validate the effectiveness of our approach, achieving over a 50-fold increase in point cloud density for constructed millimeter-wave radar environmental maps with a point cloud accuracy better than 0.1 m. This enriches the details of millimeter-wave radar environmental map construction and enhances the depth of information contained within the environmental maps. Our method supports technological advancements in path planning and scene understanding based on millimeter-wave radar environmental maps. Future research can further explore applications, such as millimeter-wave radar environmental map recognition.

## Figures and Tables

**Table 1:** Comparison of MAE metrics for millimeter-wave radar depth image reconstruction using different methods.

	Radar Frame	Radar Submap	Traditional ISP [29]	Sparsity Invariant CNN [30]	RadarHD [26]	HawkEye [20]	Proposed Method
Env. 1	1.5019	0.3935	0.2831	0.1494	0.1218	0.0968	0.1001
Env. 2	2.2103	0.4033	0.2928	0.1392	0.1163	0.0908	0.1017
Env. 3	1.0647	0.2636	0.1667	0.1340	0.0576	0.0388	0.0386
Env. 4	2.2139	0.4029	0.2934	0.1901	0.0843	0.0476	0.0521
Env. 5	1.8179	0.3299	0.2380	0.1548	0.0841	0.0576	0.0608
Env. 6	2.2416	0.4410	0.3377	0.2501	0.0981	0.0754	0.0855
Env. 7	1.9793	0.4719	0.3631	0.2155	0.1212	0.0489	0.0670

**Table 2:** Comparison of RMSE metrics for millimeter-wave radar depth image reconstruction using different methods.

	Radar Frame	Radar Submap	Traditional ISP [29]	Sparsity Invariant CNN [30]	RadarHD [26]	HawkEye [20]	Proposed Method
Env. 1	2.2706	0.4641	0.3535	0.2133	0.1904	0.1827	0.1889
Env. 2	2.2774	0.4725	0.3626	0.2141	0.1925	0.1713	0.1887
Env. 3	1.8635	0.3617	0.2490	0.2235	0.1625	0.1489	0.1431
Env. 4	2.4428	0.5329	0.4211	0.2805	0.1983	0.1467	0.1472
Env. 5	2.3851	0.4982	0.3075	0.2273	0.1463	0.1330	0.1367
Env. 6	2.6640	0.5612	0.4534	0.3308	0.2223	0.1915	0.2038
Env. 7	2.6307	0.5255	0.4174	0.2825	0.2017	0.1249	0.1277

**Table 3:** Comparison of frame rate metrics for millimeter-wave radar depth image reconstruction using different methods.

Methods	Traditional ISP [29]	Sparsity Invariant CNN [30]	RadarHD [26]	HawkEye [20]	Proposed Method
Processing Frame Rate	150 Hz	26 Hz	18 Hz	2 Hz	15 Hz

## Data Availability

https://pan.baidu.com/s/1J-L-KZ0-fy73PczT7ReTIw?pwd=6v75.

## References

[1] Yurtsever E., Lambert J., Carballo A., Takeda K. (2020). A survey of autonomous driving: Common practices and emerging technologies. IEEE Access.

[2] Placitelli A.P., Gallo L. Low-cost augmented reality systems via 3D point cloud sensors. Proceedings of the 2011 Seventh International Conference Signal Image Technology Internet-Based System.

[3] Patole S.M., Torlak M., Wang D., Ali M. (2017). Automotive Radars: A review of signal processing techniques. IEEE Signal Process. Mag..

[4] Nowok S., Kueppers S., Cetinkaya H., Schroeder M., Herschel R. Millimeter wave radar for high resolution 3D near field imaging for robotics and security scans. Proceedings of the International Radar Symposium.

[5] Cen S.H., Newman P. Radar-only ego-motion estimation in difficult settings via graph matching. Proceedings of the International Conference on Robotics and Automation.

[6] Ziyang H., Yvan P., Sen W. RadarSLAM: Radar based Large-Scale SLAM in All Weathers. Proceedings of the IEEE/RSJ International Conference on Intelligent Robots and Systems.

[7] Barjenbruch M., Kellner D., Klappstein J., Dickmann J., Dietmayer K. Joint spatial- and Doppler-based ego-motion estimation for automotive radars. Proceedings of the IEEE Intelligent Vehicles Symposium (IV).

[8] Hügler P., Grebner T., Knill C., Waldschmidt C. (2020). UAV-borne 2-D and 3-D radar-based grid mapping. IEEE Geosci. Remote Sens. Lett..

[9] Zeng Z.Y., Dang X., Li Y., Bu X., Liang X. Angular Super-Resolution Radar SLAM. Proceedings of the IEEE/RSJ International Conference on Intelligent Robots and Systems.

[10] Almalioglu Y., Turan M., Lu C.X., Trigoni N., Markham A. (2020). Milli-RIO: Ego-motion estimation with low-cost millimetre-wave radar. IEEE Sens. J..

[11] Zhuang Y., Wang B., Huai J., Li M. (2023). 4D iRIOM: 4D Imaging Radar Inertial Odometry and Mapping. IEEE Robot. Autom. Lett..

[12] Isele S.T., Fabian H.F., Marius Z. SERALOC: SLAM on semantically annotated radar point-clouds. In Proceedings of the IEEE International Intelligent Transportation Systems Conference (ITSC).

[13] Sun S.Q., Athina P.P., Vincent H.P. (2020). MIMO radar for advanced driver-assistance systems and autonomous driving: Advantages and challenges. IEEE Signal Process. Mag..

[14] Zheng L.Q., Ma Z., Zhu X., Tan B., Li S., Long K., Sun W., Chen S., Zhang L., Wan M. TJ4DRadSet: A 4D Radar Dataset for Autonomous Driving. Proceedings of the IEEE 25th International Conference on Intelligent Transportation Systems (ITSC).

[15] Zeng Z.Y., Liang X.D., Dang X.W., Li Y.L. (2023). Joint Velocity Ambiguity Resolution and Ego-Motion Estimation Method for mmWave Radar. IEEE Robot. Autom. Lett..

[16] Steiner M., Timo G., Christian W. Chirp-sequence-based imaging using a network of distributed single-channel radar sensors. Proceedings of the IEEE MTT-S International Conference on Microwaves for Intelligent Mobility (ICMIM).

[17] Tagliaferri D., Rizzi M., Tebaldini S., Nicoli M., Russo I., Mazzucco C., Monti-Guarnieri A.V., Prati C.M., Spagnolini U. Cooperative synthetic aperture radar in an urban connected car scenario. Proceedings of the IEEE International Online Symposium on Joint Communications and Sensing (JCAS).

[18] Qian K., He Z.Y., Zhang X.Y. (2020). 3D point cloud generation with millimeter-wave radar. ACM Interactive Mobile Wearable Ubiquitous Technol..

[19] Tagliaferri D., Rizzi M., Nicoli M., Tebaldini S., Russo I., Monti-Guarnieri A.V., Prati C.M., Spagnolini U. (2021). Navigation-aided automotive sar for high-resolution imaging of driving environments. IEEE Access.

[20] Guan J.F., Madani S., Jog S., Gupta S., Hassanieh H. Through fog high-resolution imaging using millimeter wave radar. Proceedings of the IEEE/CVF Conference on Computer Vision and Pattern Recognition.

[21] Sun Y., Huang Z., Zhang H., Cao Z., Xu D. 3DRIMR: 3D reconstruction and imaging via mmWave radar based on deep learning. Proceedings of the IEEE International Performance, Computing, and Communications Conference (IPCCC).

[22] Sun Y., Zhang H., Huang Z., Liu B. (2021). DeepPoint: A Deep Learning Model for 3D Reconstruction in Point Clouds via mmWave Radar. arXiv.

[23] Sun Y., Zhang H., Huang Z., Liu B. R2p: A deep learning model from mmwave radar to point cloud. Proceedings of the Artificial Neural Networks and Machine Learning–ICANN: International Conference on Artificial Neural Networks.

[24] Wang L.C., Bastian G., Carsten A. L2R GAN: LiDAR-to-radar translation. Proceedings of the Asian Conference on Computer Vision.

[25] Lu X.X., Rosa S., Zhao P., Wang B., Chen C., Stankovic J.A., Trigoni N., Markham A. See through smoke: Robust indoor mapping with low-cost mmwave radar. Proceedings of the 18th International Conference on Mobile Systems, Applications, and Services.

[26] Prabhakara A., Jin T., Das A., Bhatt G., Kumari L., Soltanaghai E., Bilmes J., Kumar S., Rowe A. (2022). High Resolution Point Clouds from mmWave Radar. arXiv.

[27] Cai P.P., Sanjib S. MilliPCD: Beyond Traditional Vision Indoor Point Cloud Generation via Handheld Millimeter-Wave Devices. Proceedings of the ACM on Interactive, Mobile, Wearable and Ubiquitous Technologies.

[28] Nguyen M.Q., Feger R., Wagner T., Stelzer A. (2023). High Angular Resolution Method Based on Deep Learning for FMCW MIMO Radar. IEEE Trans. Microw. Theory Tech..

[29] Ku J., Harakeh A., Waslander S.L. In Defense of Classical Image Processing: Fast Depth Completion on the CPU. Proceedings of the 15th Conference on Computer and Robot Vision (CRV).

[30] Uhrig J., Schneider N., Schneider L., Franke U., Brox T., Geiger A. Sparsity Invariant CNNs. Proceedings of the International Conference on 3D Vision (3DV).

[31] Hu J., Bao C., Ozay M., Fan C., Gao Q., Liu H., Lam T.L. (2023). Deep Depth Completion From Extremely Sparse Data: A Survey. IEEE Trans. Pattern Anal. Mach. Intell..

